# Non-compliance of prescriptions by the patients

**DOI:** 10.4103/0019-5545.39770

**Published:** 2008

**Authors:** C. Shamasundar

**Affiliations:** 250, 43^rd^ Cross, 9^th^ Main, 5^th^ Block, Jayanagar, Bangalore - 560 041, Karnataka, India. E-mail: drshamasundar@yahoo.com

Sir,

Your comprehensive, but brief Editorial[1] in the latest issue of *IJP* concerning the topic of non-compliance of prescriptions by the patients, often abetted by their families, addresses a problem in clinical practice which is generally neglected in our professional literature.

If you permit me, I would like to touch upon a few more etiological issues in the dimension of prevailing social values and attitudes. They are briefly described below. If there is no change in these values and attitudes, then, it is most unlikely that the problem of non-compliance will ever get remedied.

1. During my service at NIMHANS, we used to supply free drugs to poorer out-patients to last them a month or two, with instructions to come for follow-up by the time the medicines are finished. Most would turn up many months later with or without an exacerbation of symptoms. Their reasons for not continuing the medicines and following the instructions were related to their vocation: having to attend to tilling the land, sowing, weeding or harvesting, etc. Here, the reason for non-compliance is related to their life's priorities. Their daily bread claims a higher priority than their health; they are generally hardy (resilient) people who can tolerate a considerable degree of suffering. Only an excited schizophrenic or manic patient may upset their priorities. *With the exception of a few conditions, mental health is not a priority issue for them*.
2. In the last three to four decades, there have been nation-wide projects directed at eliminating tuberculosis and leprosy with free distribution of medicines, even at patients’ doorsteps. But, follow-up studies showed poor drug compliance. Many reasons were speculated and discussed. Personally, I believe this problem to be related at least partly to an attitude of complacency: attending to a life's problem only when it crosses a ‘feelable’ threshold of tolerance, only when it becomes bothersome, not giving importance to the underlying pathology. This attitude is faithfully adopted by elected representatives in the governments: attempting to deal with problems only when they become acute.
3. As mentioned in the editorial, following the prescription advice drug is related to such factors as compliance, adherence, collaboration and cooperation. The first two are affiliated to a sense of personal discipline and the last two to a sense of responsibility. In our psychosocial literature, this sense of responsibility is called ‘internal locus of control.’ But, unfortunately, different agencies for different reasons have been severely undermining this ‘internal locus of control.’ For example: (a) Under the pressure of work-load, the health profession itself has been promoting ‘pill-box’ therapy, “you take this medicine and you will be alright.” Such clinical behaviour, though un-intentionally, fosters an external locus of control, an attitude like “I will take medicines when I am ill…” (b) This attitude is further re-enforced by governmental and even WHO slogan of “right to health.” This attitude, described by Leon Kass[2], is something like “when a citizen becomes ill, it is someone else's responsibility to set things right.”
